# Social isolation, loneliness and their relationships with depressive symptoms: A population-based study

**DOI:** 10.1371/journal.pone.0182145

**Published:** 2017-08-23

**Authors:** Lixia Ge, Chun Wei Yap, Reuben Ong, Bee Hoon Heng

**Affiliations:** Health Services and Outcomes Research, National Healthcare Group Pte Ltd, Singapore, Singapore; University of West London, UNITED KINGDOM

## Abstract

**Objectives:**

To assess the relationship between various social isolation indicators and loneliness, and to examine the differential associations that social isolation indicators, loneliness have with depressive symptoms.

**Methods:**

Baseline data for 1,919 adults (aged 21 years and above) from a representative health survey in the Central region of Singapore was used for this study. The association between social isolation indicators (marital status, living arrangement, social connectedness with relatives and friends) and loneliness (the three-item UCLA Loneliness) were assessed, and their differential associations with depressive symptoms (the Patient Health Questionnaire-9) were examined using multiple linear regression, controling for relevant covariates.

**Results:**

There was significant overlap between loneliness and social isolation. Social connectedness with relatives and friends were mildly correlated with loneliness score (|r| = 0.14~0.16). Social isolation in terms of weak connectedness with relatives and with friends and loneliness were associated with depressive symptoms even after controling for age, gender, employment status and other covariates. The association of loneliness with depressive symptoms (β = 0.33) was independent of and stronger than that of any social isolation indicators (|β| = 0.00~0.07).

**Conclusions:**

The results of the study establishes a significant and unique association of different social isolation indicators and loneliness with depressive symptoms in community-dwelling adults aged 21 and above.

## Introduction

Social relationships, a fundamental and vital component of human life, have important impacts on health. While positive social relationships are protective for health, a wealth of evidence has shown that weak social relationships are associated with a wide variety of adverse health outcomes [1–3], among which depression is an important focus. Social isolation and loneliness are reflections of objective and subjective characteristics of weak social relationships [4]. Social isolation, the objective absence or near-absence of social relationships or connections, is a quantitative measure of network size, network diversity, and frequency of contact [5] and describes the extent how an individual is socially isolated. Loneliness is the extent to which the individual emotionally feels socially isolated due to unpleasant experience or unmet needs in either quantity or quality of social relationships [1]. Loneliness, which is conceptually distinct from social isolation, can occur in the presence or absence of social isolation [6,7].

Social isolation and loneliness have been individually identified to be associated with depressive symptoms in multiple studies [8–11]. Previous research among older population has identified a wide range of social isolation indicators having impacts on depression, which include being single, living alone, having a weak or small social network and infrequency of social interactions [12,13]. However, different indicators of social isolation are rarely studied together and inconsistent findings are yielded [2,14–16], making it difficult to determine which components of social isolation are more deleterious for depression. It is therefore important to incorporate different indicators of social isolation in one study. Although, the association of loneliness with depression has been less well studied than that of social isolation, evidence has shown that higher level of loneliness is consistently associated with elevated depressive symptoms across different age groups [17,18]. As majority of prior research have focused on either social isolation or loneliness only, or merging these two together as one concept, there is uncertainty in regard to which of the two plays a more important role in depression [19]. Paucity of research which distinguishes the concepts of social isolation and loneliness, has indicated that the relationship of network size with depression becomes not significant after accounting for loneliness [14,20], while loneliness, independent of social support, predicts subsequent changes in depressive symptoms [21]. Findings of prior research among older population have indicated that loneliness is more hazardous to depression than social isolation is [22,23]. However, more studies are needed in order to confirm that the relationship between loneliness and depression is different and independent of that of various social isolation indicators among adult population.

Hence, the present study was conducted to: a) assess the relationship between various indicators of social isolation (marital status, living arrangement, social connectedness with relatives and friends) and loneliness; b) analyze the additional explanatory power of social isolation indicators and loneliness in their association with depressive symptoms in a representative community-dwelling adult population.

## Methods

### Design

Data for the present study was drawn from baseline data of the Population Health Index Survey (PHI), a longitudinal household survey on the health of community-dwelling adult population (aged 21 and above) living in the Central region of Singapore. The representative sample was obtained according to the procedure described below.

### Sample and procedure

A sampling frame of residential dwelling units was constructed by matching postal codes in the National Database on Dwellings in Singapore maintained by the Department of Statistics with the list of postal codes for the Central region (which comprises of nine planning areas). A sample of 35,000 dwelling units was selected based on stratified design with proportional allocation as the sample selection technique. Dwelling units were stratified based on proportionate allocation by planning areas (nine strata). Within each planning area, a sample of dwelling units was selected proportionately from defined broad dwelling type groups (Housing and Development Board properties, condominiums and other apartments, and landed properties).

Invitation letters were sent by post to 5,350 out of the 35,000 dwelling units which were randomly selected based on the proportionate allocation by planning areas. The sample size was calculated based on the main analysis to be used for the primary study outcome and adjusted for an assumed eligible unit rate of 90%, accessible rate of 80%, response rate of 50%, and 10% dropout rate at each of the two future time points. KISH tables were subsequently used to identify one household member from each selected dwelling unit to participate in the study. By using these sampling procedures, the results should be generalizable to the group of residents represented by the individuals included in the study.

**Fig 1:**
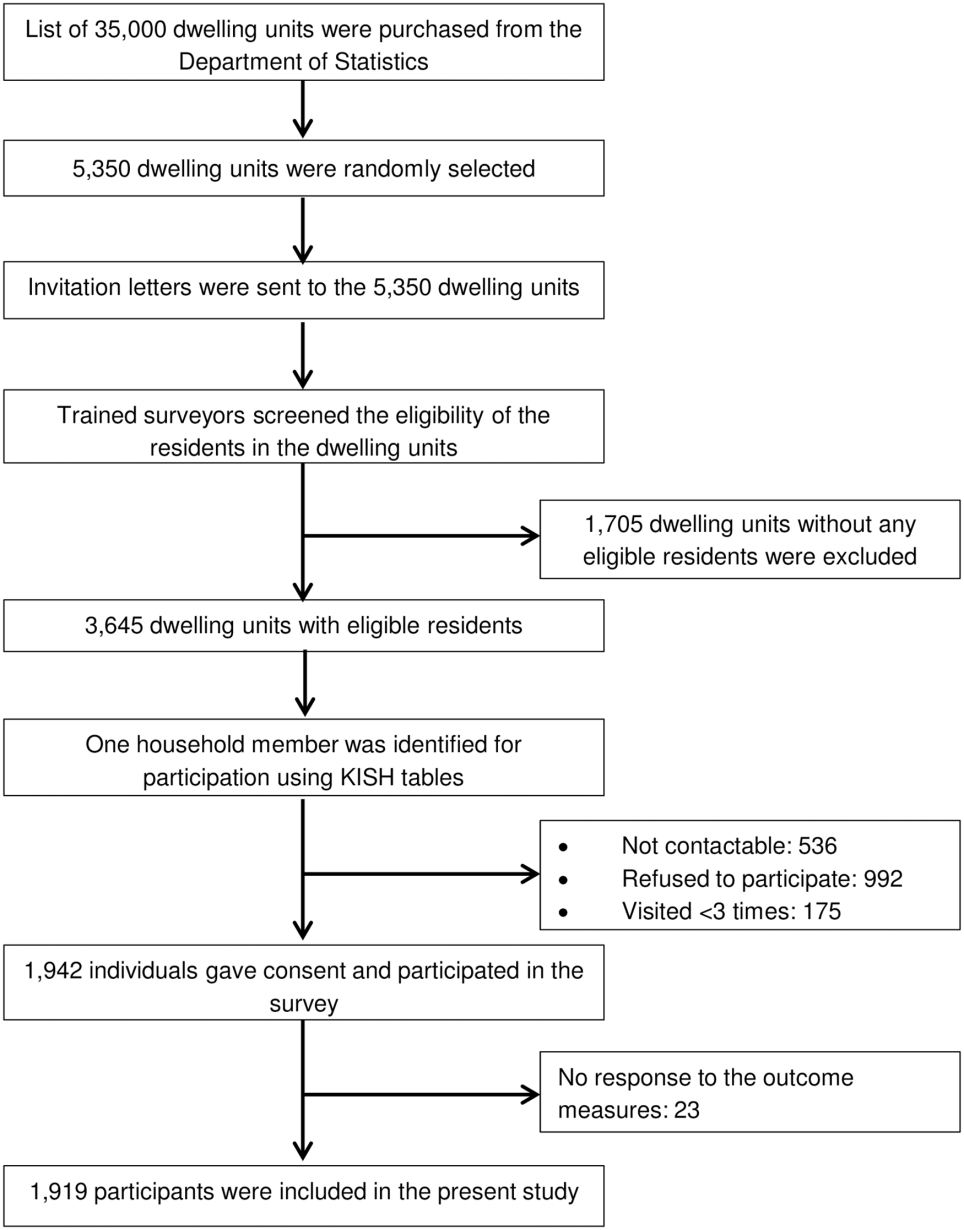
Sample design and participant selection process.

Only local citizens and permanent residents aged 21 years and above, having stayed in the selected household for more than 6 months in the past year were eligible for the survey. Between November 2015 and November 2016, trained surveyors were sent to those selected dwelling units for recruitment and conducting face-to-face survey. Units were treated as not-contactable (n = 536) if the surveyors were unable to get in touch with the selected household member after making three or more visits. A total of 1,942 individuals eventually participated in the survey (Fig 1). The response rate was 53.3%, based on a sample of 3,645 eligible residents in the selected dwelling units. The participants who did not give response to the questions about their social isolation, their perception of loneliness or depressive symptoms were excluded from the present study.

### Measures

A pre-tested, structured questionnaire consisting of social-demographics, lifestyle, medical history and a set of validated measures was used for the PHI. The following items from the PHI questionnaire were used as independent variables in the present study: socio-demographic items including age, gender, ethnicity, education, employment status and socio-economic status (money insufficiency); lifestyle related information including current smoking status and alcohol consumption. In addition, previous diagnosis of depression and self-reported diagnosis of chronic conditions (including diabetes, high blood pressure, high blood cholesterol, obesity, heart attack, heart failure, stroke or transient ischemic attacks, asthma, other chronic respiratory diseases, chronic kidney disease, cancer, arthritis or gout, osteoporosis, dementia and Parkinson’s disease) were also included.

#### Social isolation

A detailed description of the individual’s social isolation was obtained via the following three indicators: 1) marital status, 2) living arrangement, and 3) social connectedness with relatives and friends measured by the Lubben Social Network Scale-6 (LSNS-6). The LSNS-6 measures the size, closeness and frequency of contacts of a participant’s social network with reference to the level of perceived support they receive from relatives (social connected with relatives) and friends (social connected with friends). The LSNS-6 provides quantitative information on relative (extended family) and friendship ties and thus may be classed as ‘objective’ measures [24]. Each item is scored from 0 to 5. The scores for each item are added up to produce a total score of LSNS-6 ranging from 0 to 30, with lower scores indicating increased isolation. The total score of the 6 items was divided into quartiles for the analysis of the relationship between social isolation indicators and loneliness. Those in the first quartile (score ranged from 0 to12) were considered isolated, those in the second (score ranged from 13 to 16) were at high risk of isolation, those in the third (score ranged from 17 to 20) were at moderate risk of isolation, and those in the fourth (scored 21 and above) were at low risk of isolation [25]. The LSNS-6 and its two subscales have demonstrated high levels of reliability (Cronbach’s alpha = 0.80–0.89), stable factor structures, and high correlations with criterion variables [24]. The present study demonstrated good internal consistency reliability with Cronbach's alpha = 0.82.

#### Loneliness

Loneliness was assessed using the three-item UCLA Loneliness Scale [26]. This scale comprises the following three items: “*How often do you feel that you lack companionship*?*”*, “*How often do you feel left out*?*”* and “*How often do you feel isolated from others*?*”*, which are assessed on a 3-point scale (1 = hardly ever; 2 = some of the time; 3 = often). The scores for each item were added up to produce a loneliness score ranging from 3 to 9, with higher scores indicating higher loneliness levels. A loneliness score of 3–5 was classified as “not lonely” and a score of 6–9 was classified as “lonely” [27]. The UCLA Loneliness Scale has shown satisfactory reliability (Cronbach's alpha = 0.84) [28] and both concurrent and discriminant validity [26]. The internal reliability of the UCLA Loneliness Scale in this study was Cronbach's alpha = 0.85 with single factor structure explaining 81.2% of the total variance.

#### Depression

Depression was assessed using the 9-item Patient Health Questionnaire (PHQ-9). The PHQ-9 is a self-administered version of the PRIME-MD diagnostic instrument for common mental disorders. Each item of PHQ-9 is assessed on a 4-point scale (0 = not at all, 1 = several days, 2 = more than half the days, 3 = nearly every day) and the total depressive symptom score for the 9 items ranges from 0 to 27 [29]. PHQ-9 is well validated and widely used as a brief diagnostic and severity measure of depression [29]. The scale in the study demonstrated good internal consistency reliability (Cronbach's alpha = 0.77).

### Ethical considerations

The Population Health Index Survey was approved by the ethical committee of the National Healthcare Group Domain Specific Review Board (Reference Number: 2015/00269). Written informed consent was obtained from each participant after being informed about the study purpose and confidentiality of the data collected.

### Statistical analysis

Sample weights were calculated according to a three-step procedure that included the household weight, the household non-participant adjustment and the household member weight. The respective household weight for each planning area was calculated by dividing the total number of household units in the planning area by the number of sampled household units of that planning area. Adjustment for eligible units’ non-response was made to the weights to compensate for sample imbalance due to differential success in sample recruitment. Furthermore, household member weight for each participating household was made to obtain the overall sample weight.

Descriptive analyses were conducted for the sample socio-demographic characteristics. Kruskal-Wallis tests, Independent-samples Mann-Whitney U tests or Chi-square (***χ***^**2**^) tests were conducted to assess socio-demographic differences between the included and excluded samples, as well as to assess differences in socio-demographic characteristics, lifestyle and number of self-reported chronic conditions across social isolation and loneliness groups of the included sample. The distribution of social isolation indicators was obtained by extent of loneliness. Further, Spearman’s rank correlation coefficients were used to assess the correlations between LSNS-6 total score and two subscale scores, loneliness score as well as depressive symptom score. In order to investigate the independent association of social isolation and loneliness with depressive symptom score, multiple linear regression analyses were conducted with depressive symptom score as the dependent variable. The socio-demographic variables (including age, gender, ethnicity, employment status, and money insufficiency), lifestyle variables (including current smoking status and alcohol consumption), previous diagnosis of depression, and number of self-reported chronic diseases were used as covariates. Social isolation indicators including marital status, living arrangement, social connectedness with relatives and with friends (LSNS-6 family subscale and friend subscale) were added to the model to determine the incremental effect of social isolation on depressive symptom score. Subsequently, loneliness score was added to determine its incremental effect on depressive symptom score. All analyses were conducted using SPSS 18.0 software program with significant level set at 0.05.

## Results

### Sample characteristics

The final sample comprised 1,919 participants with sampling weights applied. Compared to the final sample, the excluded sample (n = 23, 19 were proxy-reported and 4 were self-reported) was significantly older (70.9±18.6 vs. 51.0±17.2, *p*<0.001), with larger proportion of Malays (26.1% vs. 7.9%, *p* = 0.011) and higher proportion of people without formal education (65.2% vs. 11.9%, *p*<0.001), mainly unemployed and inactive (91.3% vs. 36.3%, *p*<0.001), had more non-mental chronic conditions (2.9±2.3 vs. 1.1±1.4, *p*<0.001). Table 1 summarizes the socio-demographic and clinical characteristics of the final sample with sampling weights applied. Majority of the participants were married (63.8%), Chinese (78.5%), employed (63.7%) and living in HDB 3-room flat and above (84.2%). 69.8% of participants lived with spouse and/or child(ren) and 5.0% lived alone.

**Table 1:** Sample characteristics by severity of isolation and loneliness (N = 1,919).

Variable	Overall		Severity of isolation,%				*p*-value^a^	Loneliness,%		*p*-value^a^
	N	%	Isolated (n = 504)	High risk (n = 435)	Moderate risk (n = 461)	Low risk (n = 519)		Not lonely (n = 1796)	Lonely (n = 123)	
**Age, Mean**			59.3	51.4	47.0	46.3	<0.001	51.0	50.8	0.079
21–39	569	29.7	12.7	26.3	38.8	40.8		29.3	35.2	
40–59	696	36.3	35.7	41.8	34.1	34.1		36.7	29.5	
60–74	477	24.9	33.3	23.6	23.0	19.5		25.1	21.3	
≥ 75	177	9.2	18.3	8.3	4.1	5.6		8.9	13.9	
**Gender**							0.885			0.699
Male	842	43.8	44.2	43.2	45.1	42.8		43.7	45.5	
Female	1,077	56.2	55.8	56.8	54.9	57.2		56.3	54.5	
**Marital status**							<0.001			<0.001
Single	490	25.6	19.2	21.6	29.5	31.6		24.7	37.7	
Married	1,225	63.8	64.1	68.7	59.9	63.0		65.5	40.2	
Widowed/ Divorced	204	10.6	16.7	9.7	10.6	5.4		9.8	22.1	
**Ethnicity**							0.010			0.563
Chinese	1,506	78.5	76.2	79.5	81.2	77.5		78.6	77.2	
Malay	151	7.9	11.9	7.6	5.6	6.2		7.7	10.6	
Indian	213	11.1	10.1	9.9	10.8	13.3		11.2	8.9	
Others	49	2.6	1.8	3.0	2.4	3.1		2.5	3.3	
**Highest education**							<0.001			0.157
No formal education	229	11.9	26.3	10.6	4.6	5.6		11.6	17.9	
Primary	238	12.4	19.4	11.8	10.8	7.5		12.5	11.4	
Secondary (sec)	571	29.8	33.5	33.6	26.2	26.0		30.1	24.4	
Post-sec&above	881	45.9	20.8	44.0	58.4	60.9		45.9	46.3	
**Employment status**							<0.001			<0.001
Employed	1,222	63.7	50.4	64.3	72.4	68.5		64.7	48.8	
Unemployed	81	4.2	8.7	4.1	2.0	1.9		3.7	12.2	
Inactive	616	32.1	40.9	31.6	25.6	29.6		31.6	39.0	
**Living arrangement**							<0.001			<0.001
Alone	96	5.0	7.1	5.5	3.7	3.7		5.0	5.7	
With spouse, no child	275	14.3	17.5	13.3	13.2	13.1		14.9	6.5	
With child(ren), no spouse	174	9.1	14.3	7.4	10.4	4.2		8.6	16.3	
With spouse &child(ren)	890	46.4	43.1	51.7	43.7	47.5		47.4	32.5	
With others only	484	25.2	18.1	22.1	29.0	31.5		24.3	39.0	
**Money insufficiency**		14.4	30.2	12.0	10.8	4.4	<0.001	13.0	35.8	<0.001
**Smoking status**							<0.001			0.433
Never smoked	1,465	76.3	70.9	74.4	77.4	82.1		76.6	71.5	
Current smoker	240	12.5	14.5	13.4	11.1	11.2		12.4	14.6	
Former smoker	215	11.2	14.7	12.2	11.5	6.7		11.0	13.8	
**Alcohol abuse**	517	26.9	13.3	30.0	34.7	30.8	<0.001	26.9	27.0	0.981
**Number of chronic conditions, Mean**			1.6	1.0	0.9	0.7	<0.001^b^	1.0	1.7	<0.001^c^
**(SD)**			(1.7)	(1.3)	(1.3)	(1.3)		(1.4)	(2.0)	
**Diagnosis of depression**			5.0	1.6	0.6	0.8	<0.001	1.7	7.4	<0.001

The above numbers reflect weighted column %.

^a^p-value obtained using Chi-square test.

^b^p-value obtained using Kruskal-Wallis test.

^c^p-value obtained using Independent-samples Mann-Whitney U test.

### Relationship between social isolation indicators and loneliness

A total of 504 adults (26.3%) reported being isolated and 22.7% were with high risk of isolation. Adults who reported being isolated were more likely to be older, Malays, widowed or divorced, without formal education, unemployed or inactive, living alone or living with child(ren) only and current or former smoker. Besides, those who felt isolated were more likely to have more non-mental chronic conditions and previously diagnosed depression but less likely to be with alcohol abuse (Table 1).

More than 80% of the sample reported a cumulative score of three for the UCLA Loneliness Scale. There were 128 adults (6.4%) reported feeling lonely. Notably, adults who reported feeling lonely were more likely to be widowed or divorced and unemployed, more likely to have more non-mental chronic conditions and previously diagnosed depression. Although the proportion of participants who reported feeling lonely was slightly higher in the age groups of 21–39 (35.2% vs. 29.3%) and 75 and above (13.9% vs. 8.9%) compared to those who didn’t report feeling lonely, the difference was not significant (Table 1). Furthermore, there was no significant difference in gender, ethnicity, highest education, smoking status and alcohol consumption.

There was significant overlap between loneliness and social isolation indicators: while 24.7% of those who reported not lonely were in the isolated group, this proportion was 50.0% among those who reported feeling lonely (Table 2). The proportion of participants living with spouse and child(ren) who reported feeling lonely (32.8%) was lower compared to the proportion of those who did not (47.4%). Similarly, the percentage of married participants who reported feeling lonely (40.2%) was lower than that of those who did not report feeling lonely (65.5%). The correlation between social connectedness with relatives, social connectedness with friends and loneliness using Spearman’s rank correlation coefficients (Table 3) showed that there was weak correlation between loneliness and social connectedness with relatives (LSNS-6 family subscale) or with friends (LSNS-6 friend subscale) (|r| = 0.14, *p*<0.001).

**Table 2:** Distribution of social isolation indicators by extent of loneliness.

		Not lonely (n = 1,796)		Lonely (n = 123)	
		n	%	n	%
**Marital status**	Single	444	24.7	46	37.7
	Married	1,176	65.5	49	40.2
	Widowed/Divorced	176	9.8	27	22.1
**Living arrangement**	live alone	89	5.0	7	5.7
	with spouse, no child	267	14.9	8	6.6
	with child(ren), no spouse	154	8.6	20	16.4
	with spouse and child(ren)	850	47.4	40	32.8
	with others only	436	24.3	48	38.5
**LSNS-6**	Isolated	443	24.7	61	50.0
	High risk	405	22.5	30	24.6
	Moderate risk	443	24.7	18	14.8
	Low risk	506	28.2	13	10.7

The above percentages reflected weighted column %.

**Table 3:** Correlation matrix (95% CI), means and standard deviations of continuous social isolation indicators, loneliness and depressive symptom score (N = 1,919).

	Social connectedness with relatives	Social connectedness with friends	LSNS-6 social isolation	loneliness	Depressive symptom score
**Social connectedness with relatives**	1	-	-	-	-
**Social connectedness with friends**	0.45	1	-	-	-
	(0.41, 0.48)				
**LSNS-6 social isolation**	0.79	0.89	1	-	-
	(0.78, 0.81)	(0.88, 0.90)			
**loneliness**	-0.14	-0.14	-0.16	1	-
	(-0.19, -0.10)	(-0.18, -0.10)	(-0.21, -0.12)		
**Depressive symptom score**	-0.15	-0.13	-0.16	0.32	1
	(-0.19, -0.10)	(-0.18, -0.09)	(-0.21, -0.12)	(0.27, 0.36)	
**Mean ± SD**	9.0 ± 3.2	7.5 ± 4.0	16.4 ± 6.1	3.4 ± 1.0	1.1 ± 2.4

p-values for all the correlation coefficients were <0.001.

### Association of social isolation indicators and loneliness with depressive symptoms

As shown in Table 3, the correlation between social connectedness with relatives or with friends and depressive symptom score was low (|r| = 0.13~0.15, *p*<0.001), whereas the correlation between loneliness and depressive symptom score was moderate (r = 0.32, *p*<0.001). The comparison of depressive symptom score among isolation and loneliness groups showed that those who were more isolated had higher depressive symptom score (mean: 3.5 for isolated group vs. 1.4 for low risk group) and those who felt lonely also had higher depressive symptom score (mean: 5.0) than those who did not feel lonely (mean: 2.0).

The multiple linear regression analyses were shown in Table 4. Social isolation indicators explained 2.4% of the variation in depressive symptom score, and this change in R^2^ was statistically significant. A larger increase in the percentage of variance explained (9.7%) was observed when loneliness score was added to the model.

**Table 4:** Linear regression analyses for depressive symptoms.

Variables	Coefficients		
	Regression 1	Regression 2	Regression 3
Age	-0.03	-0.05	-0.01
Female (Ref. Male)	0.07**	0.06**	0.06**
Ethnicity (Ref. Chinese)			
Malay	0.03	0.02	0.02
Indian	0.00	0.01	0.01
Others	0.05*	0.05*	0.03
Employment status (Ref. Employed)			
Unemployed	0.15***	0.12***	0.09***
Inactive	0.01	0.00	-0.01
Money insufficiency	0.16***	0.13***	0.10***
Currently smoking (Ref. not smoking)	0.01	0.01	0.01
Alcohol abuse	0.08***	0.08**	0.07**
Previous diagnosis of depression	0.23***	0.22***	0.19***
Number of chronic conditions	0.18***	0.17***	0.14***
Marital status (Ref. Single)			
Married		-0.11	-0.06
Widowed /Divorced		0.01	0.00
Living arrangement (Ref. With spouse & child(ren))			
Alone		-0.07*	-0.05
With spouse, no child		0.01	0.01
With child(ren), no spouse		-0.01	0.00
With others only		-0.04	-0.02
Social connectedness with relatives		-0.06*	-0.04
Social connectedness with friends		-0.11***	-0.07**
Loneliness			0.33***
R^2^	0.18	0.20	0.30
ΔR^2^	-	0.02	0.10

* p<0.05.

**p<0.01.

***p<0.001.

Among the indicators of social isolation, only social connectedness with relatives and with friends had significant association with depressive symptoms. While the association between social connectedness with relatives and depressive symptoms became non-significant after loneliness was introduced, the association between social connectedness with friends and depressive symptom score remained significant. Other indicators including marital status and living arrangement didn’t show significant association with depressive symptoms. A higher score of loneliness was significantly associated with higher depressive symptom score, which is independent of the association between any social isolation indicators and depressive symptoms.

## Discussion

Social isolation and loneliness are growing problems among the ageing society. The comparability of the distribution of social isolation in our sample with another study [30] among community-dwelling elderly in Singapore using the same measure indicates that although social isolation is most common in elder population, their prevalence among the young and middle-aged group are also of concern. The importance of high frequencies of social connectedness with relatives on low level of loneliness has been recognized in prior literature [31], our data shows that the social connectedness with friends also has a statistically significant association with loneliness.

The present study shows that depressive symptom score is higher among more socially isolated participants. Although various studies have reported that social isolation (with definition and measurement varied) is associated with depressive symptoms, the associations of social connectedness with relatives and friends with depressive symptoms are rarely distinguished. This study disentangles these two aspects of social isolation and examines their associations (together with other social isolation indicators) with depressive symptoms respectively. The results show that poorer social connectedness with friends and with relatives are associated with elevated depressive symptoms, even after controlling for socio-demographic factors, current smoking status, alcohol consumption, previous diagnosis of depression and number of chronic conditions. Furthermore, our data indicates that the effect of social connectedness with friends on depressive symptoms is more predominant than that of social connectedness with relatives among the study population. This finding confirms the importance of friendship in preventing or alleviating depressive symptoms. Previous study indicates that living alone contributes to poorer depression among elderly persons [13,22,32], however, our study suggests that living alone does not appear to have a significant association with depressive symptoms in adult population. This implies that social isolation is not simply a function of the amount or format of social connectedness (i.e. living alone), other aspects of social isolation (i.e. social support and social integration) may have higher impact on depressive symptoms in adult population.

On the other hand, similar to the findings from both local [22] and some international studies [8,33,34], those who report higher loneliness score have elevated depressive symptoms, even after controlling for socio-demographic factors and other relevant covariates. The influence of loneliness on depressive symptoms is independent of that of any social isolation indicators. Furthermore, compared to social isolation, loneliness has a much stronger association with depressive symptoms in adults aged 21 and above.

The weak correlation between social isolation indicators and loneliness reflects the differences of these two concepts, which has been indicated in other studies [27,28]. Our analysis on the association of the interaction of social isolation and loneliness with depressive symptoms (results were not reported in this paper) indicates that the strength of the relationship between social isolation and depressive symptoms varies by loneliness.

The strengths of this study includes the use of a sample of representative adult population in which it is possible to control for multiple demographic and health indicators. In addition, we analyze the associations between different indicators of social isolation and loneliness as well as the differential association that social isolation indicators and loneliness have with depressive symptoms. The findings of this study confirm that social isolation and loneliness are independently associated with depressive symptoms and loneliness has a stronger association with depressive symptoms than social isolation. In addition, within the concept of social isolation, social connectedness with relatives and with friends, rather than marital status or living arrangement, plays a predominant role in the association with depressive symptoms. Nonetheless, the cross-sectional nature of the present observational study limits the claims of causal inferences; hence we cannot conclude that an increase in the severity of social isolation or loneliness causes increases in depressive symptom score or vice versa. The subsequent follow-up of the same participants at 1-year and 2-year may provide some value in identifying the possible causation of changes in depressive symptoms by changes in social isolation and loneliness.

## Supporting information

S1 File The questionnaire used in the present study. (PDF)
