# Supplementary material for: Social isolation, loneliness and their relationships with depressive symptoms: A population-based study
Source: PLoS One. 2017 Aug 23;12(8):e0182145. doi: 10.1371/journal.pone.0182145 (PMC5568112; doi:10.1371/journal.pone.0182145)
Supplement: S1 File — (PDF) [file pone.0182145.s001.pdf]

Reference No. ☐☐☐☐-☐☐

National Healthcare Group
Adding years of healthy life

Population Health Index Survey

Date of interview : ____________________

Reference No. ☐☐☐☐-☐☐ Self-reported ☐ Proxy* ☐

*The proxy is to rate how he/she (the proxy) thinks the respondent would rate his/her own responses if he/she (the respondent) was able to communicate it.

Demographics

Age : __________

Gender :

☐ 1) Male

☐ 2) Female

Marital status :

1) Single	2) Married	3) Cohabiting	4) Widowed	5) Divorced / Separated
☐	☐	☐	☐	☐

Ethnicity :

☐ 1) Chinese

☐ 2) Malay

☐ 3) Indian

☐ 4) Eurasian

☐ 5) Others: ____________________

Employment status :

1) Employed: ☐Full-time ☐Part-time

2) Unemployed: ☐Voluntary ☐Involuntary

3) Inactive: ☐Homemaker ☐ Retired

☐Non-paid ☐Student / National Service

4) Permanently sick/unfit for work

Highest Education Attained :

- ☐ 0) No formal qualification/Pre-Primary/Lower Primary
  (Primary education without PSLE/PSPE or equivalent; Certificate in BEST 1-3)
- ☐ 1) Primary
  (Certificate in BEST 4; PSLE/PSPE or equivalent; at least 3 WSQ Statements of attainment in WPLN at Level 1 or 2)
- ☐ 2) Lower Secondary
  (Secondary education without a GCE 'O'/'N' Level pass or their equivalent; Certificate in WISE 1-3; Basic vocational certificates; at least 3 WSQ Statements of attainment in WPLN at Level 3 or 4)
- ☐ 3) Secondary
  ('N' Level; 'O' Level; Nitec (intermediate) or equivalent; ISC or equivalent; at least 3 WSQ Statements of attainment in WPLN at Level 5 and above)
- ☐ 4) Post-Secondary
  ('A'/'H2' Level / Nitec or equivalent/WSQ Certificate or equivalent)
- ☐ 5) Polytechnic Diploma
- ☐ 6) Professional Qualification and other diploma
  (ITE/NIE/SIM/LaSalle-SIA/NAFA/WSQ diploma or equivalent)
- ☐ 7) Bachelor's Degree or equivalent
- ☐ 8) Postgraduate Diploma/ Certificate
- ☐ 9) Master's and Doctorate or equivalent

Living arrangement :

- ☐ 1) Alone
- ☐ 2) With spouse
- ☐ 3) With children or grandchildren
- ☐ 4) With other relatives or friends
- ☐ 5) With other unrelated individuals: ____________________

Medical history

1.	Have you ever been told to have any of these conditions by a Western-trained doctor?	0) No	1) Yes	2) Do not know
a)	Diabetes / gestational diabetes	☐	☐	☐
b)	High blood pressure	☐	☐	☐
c)	High blood cholesterol	☐	☐	☐
d)	Overweight / obesity	☐	☐	☐
e)	Heart attack	☐	☐	☐
f)	Heart failure	☐	☐	☐
g)	Stroke / Transient Ischaemic attacks	☐	☐	☐
h)	Asthma	☐	☐	☐
i)	Chronic bronchitis / emphysema / COPD	☐	☐	☐
j)	Chronic kidney disease	☐	☐	☐
k)	Cancer (Please specify:______________________)	☐	☐	☐
l)	Osteoarthritis / gout / rheumatoid arthritis	☐	☐	☐
m)	Osteoporosis	☐	☐	☐
n)	Depression	☐	☐	☐
o)	Anxiety disorder	☐	☐	☐
p)	Dementia / Alzheimer's	☐	☐	☐
q)	Schizophrenia	☐	☐	☐
r)	Parkinson's disease	☐	☐	☐

Lifestyle

		0) No	1) Yes	If yes, how long would 1 pack (20 sticks) last you?
2.	Do you currently smoke tobacco products?	☐	☐	____________________ days

	0) Never	1) Monthly or less	2) 2-4 times a month	3)2-3 times a week	4) ≥ 4 times a week
**3. How often do you have a drink[1] containing alcohol?**	☐	☐	☐	☐	☐

	0) 1 or 2	1) 3 or 4	2) 5 or 6	3) 7 to 9	4) 10 or more
**4. How many drinks[1] containing alcohol do you have on a typical day when you are drinking?**	☐	☐	☐	☐	☐

	0) Never	1) Less than monthly	2) Monthly	3) 2-3 times per week	4) ≥ 4 times a week
**5. How often do you have six or more drinks[1] on one occasion?**	☐	☐	☐	☐	☐

[1] *One alcoholic drink refers to 1 can/small bottle (~285mls) of beer or 1 glass (~120mls) of wine or 1 measure (~30mls) of spirits*

Mental health

Patient Health Questionnaire

		0)Not at all	1)Several days	2)More than half the days	3)Nearly every day
6.	**Over the <u>last 2 weeks</u>, how often have you been bothered by any of the following problems?**				
	a. Little interest or pleasure in doing things	☐	☐	☐	☐
	b. Feeling down, depressed, or hopeless	☐	☐	☐	☐
	c. Trouble falling or staying asleep, or sleeping too much	☐	☐	☐	☐
	d. Feeling tired or having little energy	☐	☐	☐	☐
	e. Poor appetite or overeating	☐	☐	☐	☐
	f. Feeling bad about yourself — or that you are a failure or have let yourself or your family down	☐	☐	☐	☐
	g. Trouble concentrating on things, such as reading the newspaper or watching television	☐	☐	☐	☐
	h. Moving or speaking so slowly that other people could have noticed Or the opposite — being so fidgety or restless that you have been moving around a lot more than usual	☐	☐	☐	☐
	i. Thoughts that you would be better off dead or of hurting yourself in some way	☐	☐	☐	☐

Socio-economic status

		0) No	1) Yes
7.	**Do you often run out of money, even with proper spending plan, to buy essential items or pay bills to maintain basic living needs?** (i.e. Accommodation, food, transportation and healthcare)?	☐	☐

Social

Social isolation

		0) 0	1) 1	2) 2	3) 3-4	4) 5-8	5) ≥ 9
8.	**How many relatives/friends do you see/hear from at least once a month?**						
a)	Relatives (including children, grandchildren, in-laws, siblings, cousins, etc.)	☐	☐	☐	☐	☐	☐
b)	Friends	☐	☐	☐	☐	☐	☐

		0) 0	1) 1	2) 2	3) 3-4	4) 5-8	5) ≥ 9
9.	**How many relatives/friends do you feel at ease with whom you can talk about private matters?**						
a)	Relatives (including children, grandchildren, in-laws, siblings, cousins, etc.)	☐	☐	☐	☐	☐	☐
b)	Friends	☐	☐	☐	☐	☐	☐

		0) 0	1) 1	2) 2	3) 3-4	4) 5-8	5) ≥ 9
10.	**How many relatives/friends do you feel close to such that you could call on them for help?**						
a)	Relatives (including children, grandchildren, in-laws, siblings, cousins, etc.)	☐	☐	☐	☐	☐	☐
b)	Friends	☐	☐	☐	☐	☐	☐

Loneliness

		1) Hardly ever	2) Some of the time	3) Often
11.	**How often do you feel that you lack companionship?**	☐	☐	☐
12.	**How often do you feel left out?**	☐	☐	☐
13.	**How often do you feel isolated from others?**	☐	☐	☐
